# ThermoBase: A database of the phylogeny and physiology of thermophilic and hyperthermophilic organisms

**DOI:** 10.1371/journal.pone.0268253

**Published:** 2022-05-10

**Authors:** Juliana DiGiacomo, Christopher McKay, Alfonso Davila

**Affiliations:** NASA Ames Research Center, Moffett Field, California, United States of America; University of Utah, UNITED STATES

## Abstract

Thermophiles and hyperthermophiles are those organisms which grow at high temperature (> 40°C). The unusual properties of these organisms have received interest in multiple fields of biological research, and have found applications in biotechnology, especially in industrial processes. However, there are few listings of thermophilic and hyperthermophilic organisms and their relevant environmental and physiological data. Such repositories can be used to standardize definitions of thermophile and hyperthermophile limits and tolerances and would mitigate the need for extracting organism data from diverse literature sources across multiple, sometimes loosely related, research fields. Therefore, we have developed ThermoBase, a web-based and freely available database which currently houses comprehensive descriptions for 1238 thermophilic or hyperthermophilic organisms. ThermoBase reports taxonomic, metabolic, environmental, experimental, and physiological information in addition to literature resources. This includes parameters such as coupling ions for chemiosmosis, optimal pH and range, optimal temperature and range, optimal pressure, and optimal salinity. The database interface allows for search features and sorting of parameters. As such, it is the goal of ThermoBase to facilitate and expedite hypothesis generation, literature research, and understanding relating to thermophiles and hyperthermophiles within the scientific community in an accessible and centralized repository. ThermoBase is freely available online at the Astrobiology Habitable Environments Database (AHED; https://ahed.nasa.gov), at the Database Center for Life Science (TogoDB; http://togodb.org/db/thermobase), and in the S1 File.

## Introduction

Thermophiles and hyperthermophiles constitute two sub-classes of extremophile microorganisms which grow optimally above high (40–50°C) and extremely high (70–80°C) temperatures, respectively [1]. Phylogenetic and physiological evidence suggests an extremely ancient origin for these organisms which parallels the origins or life and the last universal common ancestor (LUCA) [2, 3]. In both the Archaeal and Bacterial domains, phylogenetic analysis places most hyperthermophiles and a disproportionate number of thermophiles near their evolutionary nodes. Since the development of the earliest phylogenetic trees, which genetically discriminated between the three domains of life based upon the small subunit ribosomal RNA gene, it has been acknowledged that the earliest branches comprising the Bacteria and Archaea are populated by thermophiles [4]. This observation has been confirmed with further reconstructions of the tree of life [2, 5, 6].

The mechanisms of physiological and genetic adaptation to high temperature continues to be a subject of research and multiple factors have been proposed to contribute to high temperature tolerance, including membrane composition, membrane bioenergetics, or the thermal stability of enzymes, DNA and RNA [7]. Research into the origin and nature of thermophiles and hyperthermophiles also has applications for understanding the origin and evolution of life on Earth and the possibility of life on other planetary bodies- the study of Astrobiology. The ancient nature of thermophiles and hyperthermophiles aligns with theories that chemical disequilibria in hydrothermal vents could have provided a geochemical template for the evolution of the first metabolic networks [3]. Modern hydrothermal vents host rich ecosystems in which thermophiles and hyperthermophiles maintain the ability to generate thermodynamic disequilibrium through the reduction of locally available sulfur, iron, and carbon dioxide [3]. Sulfur and iron are of particular interest considering these species would have been water soluble when Earth’s atmosphere was anoxic at the time of the hypothesized origin of life [3]. More recently, thermophiles and hyperthermophiles have become a biological analog for possible forms of life in extraterrestrial environments, especially the subsurface oceans of the icy moons of the outer solar system such as Europa or Enceladus. Hydrothermal activity similar to that found in Earth’s oceans appears to also be prevalent in these Ocean Worlds, representing an engine of chemical disequilibrium and a source of redox energy and nutrients that life could exploit, akin to terrestrial thermophilic methanogenic archaea, which currently hold the record for growth at the highest temperature [8].

Thermophiles possess biological adaptations which allow the organism and its component parts to function and remain stable in otherwise inhospitable environments. In biotechnology, crucial methods such as Polymerase Chain Reaction (PCR) require the functionalities of polymerase from *Pyrococcus furiosus* (Pfu-Pol) or *Thermophilus aquaticus* (Taq-Pol) [9]. Such thermotolerant polymerases significantly increased the efficiency of this DNA replication method as it allowed for multiple thermal cycles without the need for constant replenishing of the denatured enzyme. RNA and DNA ligases from such heat tolerant organisms also have applications in such integral methods as Gibson assembly (Tag-ligase), Ligase Chain Reaction, and construction of mRNA sequencing libraries where enzymes not only function at high temperature but are also able to ligate combinations of blunt and cohesive ends [9].

Industrial processes have also capitalized on the ability for enzymes from thermophiles and hyperthermophiles to function at high temperatures. The sugar industry, paper industry, fruit industry, starch processing, food processing, alcohol production, lactose-free milk production, animal feed production, and laundry detergents are just a fraction of the industrial applications for thermostable enzymes [10]. Such enzymes pose several advantages over their mesophilic counterparts where efficacy at high temperatures reduces the risk of contamination and viscosity of the reaction medium, while increasing bioavailability and solubility of organic compounds, and the diffusion coefficient and concentrations of substrates and products allowing for high reaction rates [11]. The production and diversity of these thermostable enzymes has continued to grow in recent years with advances in isolation methods of thermophilic Archaea, Bacteria, and Eukarya from different ecological niches [11].

Outside of isolated thermostable enzymes, the metabolic activities of thermophiles and hyperthermophiles also have applications in biotechnology and industrial processes. Directly, the ancient metabolisms which are utilized by most thermophilic archaea have been re-directed to produce simple C1 compounds such as carbon monoxide, formate, carbon dioxide, and methane, molecular hydrogen, and reduced/oxidized sulfur or transition metals [9]. However, these organisms also have metabolic applications for the bioconversion of xylose to ethanol (*Thermoanaerobacter ethanolicus*), crude oil degradation (*Bacillus sp*.), heavy metal recovery (*Bacillus sp*.), keratin degradation (*Fervidobacterium pennavorans*), saccharification of agricultural residues (*Sporotrichum thermophile*), hygiene indication in dairy products (*Anoxybacillus flavithermus*), remediation of textile dyes (*Geobacillus thermocatenulatus*), and even breast cancer treatments (*Aspergillus terreus*) [10].

The efficiency of scientific and technological investigation benefits from access to consolidated databases of relevant organism information. However, the generation of a database of thermophiles is especially difficult, rooted in the fact that thermophiles and hyperthermophiles are difficult to identify in a broad search. Primarily, this is because there is no universal definition for thermophiles or hyperthermophiles. Various sources posit differing minimum optimal temperatures for thermophilic classification. This value can range from 40°C, 45°C, or 50°C [12–14]. The cut off for hyperthermophiles is similarly unclear, described as 70°C, 75°C, or 80°C [12–14]. Due to the ambiguous nature of these definitions, classification of an organism’s identity as a mesophile, thermophile or hyperthermophile can oftentimes conflict in published investigations. This makes it difficult to even identify thermophiles or hyperthermophiles accurately without referencing to the original literature resources which characterize optimal growth temperature. Besides this linguistic hurdle, the task of collecting additional environmental and physiological data poses an even larger expenditure of labor and time.

Over the past 10 years, several repositories have been developed in order to centralize and enhance understandings of thermophiles in the context of other extremophiles [15–17]. TEMPURA is an online database which reports extreme growth temperatures in Prokaryotes [15]. ExProtDB is a database of extremophilic proteins and their host organisms with 259 relevant thermophiles identified [16]. ExtremeDB is a now inaccessible repository which once housed the general characteristics and genomic information for 865 extremophiles including 310 thermophiles [17]. Our database builds on these efforts by including extensive physiological and environmental data for all currently identified thermophiles.

ThermoBase was developed as a centralized database to facilitate the retrieval of primary environmental and physiological data pertaining to thermophile and hyperthermophiles. This freely available repository currently hosts 1238 thermophiles and hyperthermophiles species from the three domains of life. ThermoBase reports taxonomic, metabolic, environmental, and physiological information in addition to literature resources. This includes parameters such as ions for chemiosmosis, optimal pH and range, optimal temperature and range, optimal pressure, and optimal salinity. We utilize a lower-limit definition for thermophilicity (average optimal growth temperature ≥40°C) to allow for custom nomenclature definition preferences to be applied by the user. Therefore, with this 40°C limit, we include all known organisms which could possibly be considered thermophilic. The database format also allows for search features and sorting of other qualitative parameters. It is the goal of ThermoBase to facilitate and expedite hypothesis generation, literature research, and understanding relating to thermophiles and hyperthermophiles within the scientific community in an accessible and centralized repository.

## Methods

ThermoBase was constructed from an initial database sourced from the work of Campbell et al. [19]. This work distinguished between psychrophilic, mesophilic, thermophilic, and hyperthermophilic species from the 7079 finished genomes from the Joint Genome Institute for the purpose of classifying ferredoxin and flavodoxin proteins [18]. Some accessible metadata, including average pH, average temperature, ecosystem, energy source, oxygen requirement, and taxonomic data, was available in certain cases using Joint Genome Institute metadata labels.

The original dataset was then filtered to only include species specified as thermophiles or hyperthermophiles. However, the exact quantitative limits for these labels were not specified and had to be independently confirmed. The original metadata was also largely incomplete (<150 relevant species) and did not include all species nor desired parameters. ThermoBase was then expanded through a deep literature search of thermophilic and hyperthermophilic archaea, bacteria, and Eukarya in order to incorporate all appropriate species and available metadata. This required reference to multiple sources per species in order to collect data for taxonomic information, genome ID, general ecosystem, environment, energy source, metabolism, ions for chemiosmosis, oxygen requirement, optimal pH and range, optimal temperature and range, pressure at optimal temperature, optimal pressure, and optimal salinity.

ThermoBase was made available in various formats. The original file is downloadable as an Excel Spreadsheet from the NASA Astrobiology Habitable Environments Database (AHED; https://ahed.nasa.gov) and in the S1 File. ThermoBase is also available as an online database developed using TogoDB, a database hosting service by the Database Center for Life Science. The original database file (comma-separated values file) was uploaded to the hosting service and formatted for proper integration. The back-end data is stored at a SPARQL endpoint (http://togodb.org/sparql/thermobase). The user interface was then customized and reformatted using HTML (HyperText Mark-up Language).

## Results and discussion

ThermoBase Version 1.0 hosts detailed physiological and environmental descriptions of 1238 thermophilic and hyperthermophilic species with optimal growth temperatures equal to or above 40°C. The database encompasses all three domains of life with 373 archaeal, 836 bacterial, and 28 eukaryal species from over 226 distinct taxonomic families. Parameters for species metadata include generic information such as taxonomic information, genome ID, general ecosystem, environment, energy source, metabolism, and oxygen requirement as well as more specific categorizations such as ions for chemiosmosis, optimal pH and range, optimal temperature and range, pressure at optimal temperature, optimal pressure, and optimal salinity. Each entry also includes a key source for further investigation of a particular species. The total compiling of the database referenced over 590 unique scientific studies from 1967 to 2020 in an extensive literature search over the course of 11 months. Although all parameters could not be populated for all species, ThermoBase version 1.0 represents the extent of peer reviewed data which was available as of March 2022.

ThermoBase as a web-based service also allows for the searching of key words or values and sorting of its extensive qualitative and quantitative species data. In its Microsoft Excel Worksheet format, standard spreadsheet processing tools allow for users to reorganize and group single or multiple parameters at a time using sorting, custom sorting, and sorting level functionalities. Basic or advanced data filters can also be applied singly or cumulatively to isolate and stratify certain parameters or search terms of interest. These tools allow the user to focus their navigation of this extensive database for more efficient hypothesis generation, literature research, and understanding relating to thermophiles and hyperthermophiles within the scientific community and beyond.

The data parameters described by ThermoBase have immediate applications toward several major inquiries within studies of the limits of life, particularly the investigation of the relationship between heat tolerance and other taxonomic, physiological, or environmental properties. Initial analyses of the database confirm that heat tolerance is phylogenetically widespread and involves diverse biochemical and physiological adaptations, with H^+^/Na^+^ bioenergetics and metabolism playing a significant role [19]. In addition, when organisms sorted by their maximum temperature, it is interesting that the top 50 species are exclusively within the domain of Archaea- those that have a maximum growth temperature >95°C. Within this group strict anaerobes dominate but a few facultative anaerobes are present. While a methanogen is found to function at the highest temperature, within the top 50 hyperthermophiles, sulfur reduction is the most common metabolic strategy. This is interesting to note as by some metrics there is about the same amount of research on methanogen hyperthermophiles as on sulfur reducing hyperthermophiles–based on a simple web search on the terms “hyperthermophiles methanogens” and “hyperthermophiles sulfur reducers”. The structure of ThermoBase allows for such observations to be ascertained with the potential to spark new academic research.

ThermoBase is freely available online at the NASA Astrobiology Habitable Environments Database (AHED; https://ahed.nasa.gov) and at the Database Center for Life Science (TogoDB; http://togodb.org/db/thermobase), and in the S1 File. The ThermoBase online database continues to be manually updated with new scientific publications and user contributions.

## Supporting information

S1 File ThermoBase_ver_1.0_2022. A local spreadsheet of the ThermoBase database which was updated as of March 2022.
(XLSX)
